# Self-Assembly of pH-Labile Polymer Nanoparticles for Paclitaxel Prodrug Delivery: Formulation, Characterization, and Evaluation

**DOI:** 10.3390/ijms21239292

**Published:** 2020-12-05

**Authors:** Shani L. Levit, Narendar Reddy Gade, Thomas D. Roper, Hu Yang, Christina Tang

**Affiliations:** 1 Chemical and Life Science Engineering Department, Virginia Commonwealth University, Richmond, VA 23284, USA; levitsl@vcu.edu (S.L.L.); g.narendarreddy@gmail.com (N.R.G.); tdroper@vcu.edu (T.D.R.); hyang2@vcu.edu (H.Y.); 2 Department of Pharmaceutics, Virginia Commonwealth University, Richmond, VA 23298, USA; 3 Massey Cancer Center, Virginia Commonwealth University, Richmond, VA 23298, USA

**Keywords:** nanoparticles, ovarian cancer, paclitaxel, self-assembly, formulation, prodrug, polymer, micelle, polyphenol, drug delivery

## Abstract

The efficacy of paclitaxel (PTX) is limited due to its poor solubility, poor bioavailability, and acquired drug resistance mechanisms. Designing paclitaxel prodrugs can improve its anticancer activity and enable formulation of nanoparticles. Overall, the aim of this work is to improve the potency of paclitaxel with prodrug synthesis, nanoparticle formation, and synergistic formulation with lapatinib. Specifically, we improve potency of paclitaxel by conjugating it to α-tocopherol (vitamin E) to produce a hydrophobic prodrug (Pro); this increase in potency is indicated by the 8-fold decrease in half maximal inhibitory concentration (IC_50_) concentration in ovarian cancer cell line, OVCA-432, used as a model system. The efficacy of the paclitaxel prodrug was further enhanced by encapsulation into pH-labile nanoparticles using Flash NanoPrecipitation (FNP), a rapid, polymer directed self-assembly method. There was an 1100-fold decrease in IC_50_ concentration upon formulating the prodrug into nanoparticles. Notably, the prodrug formulations were 5-fold more potent than paclitaxel nanoparticles. Finally, the cytotoxic effects were further enhanced by co-encapsulating the prodrug with lapatinib (LAP). Formulating the drug combination resulted in synergistic interactions as indicated by the combination index (CI) of 0.51. Overall, these results demonstrate this prodrug combined with nanoparticle formulation and combination therapy is a promising approach for enhancing paclitaxel potency.

## 1. Introduction

Taxol, a formulation of paclitaxel (PTX), is widely used for treating ovarian carcinomas. However, there are challenges associated with taxol treatment due to its poor solubility, poor bioavailability, and acquired drug resistance mechanisms that together result in low drug efficacy [1,2,3,4]. Design of paclitaxel prodrugs is a promising method to improve its anticancer activity [5,6]. Prodrug design involves conjugation via a degradable linkage to retain therapeutic activity. The conjugation chemistry [5,6] can be selected to tune release half-life and release mechanism and has been reviewed elsewhere [7]. Overall, the aim of this work is to improve the potency of paclitaxel though a combination of prodrug synthesis, nanoparticle formation, and formulation with lapatinib (LAP) with synergistic effects. 

Hydrophobic modification of paclitaxel can enable formulation of nanoparticles with longer circulation half-lives [6]. For example, Pustulka, et al. encapsulated paclitaxel and paclitaxel-silicate prodrugs with various hydrophobicities using Flash NanoPrecipitation (FNP). This Flash NanoPrecipitation method is promising for rapid, scalable production of nanoparticle formulations [8]. Their work demonstrates that rapid formulation of paclitaxel prodrugs is possible using Flash NanoPrecipitation; however, no evaluation of drug potency was included.

Other work has demonstrated that nanoparticle formulations of paclitaxel prodrugs can improve drug potency. For example, poly(ethylene glycol)-b-poly(acrylic acid) (PEG-b-PAA) conjugated paclitaxel self-assembled into micelles. The prodrug was encapsulated into pH-responsive micelles and rapid drug release was achieved under acidic conditions. The micelle platform resulted in a 5-fold increase in drug potency compared to paclitaxel as indicated by the decrease in the half maximal inhibitory concentration (IC_50_) value in lung and cervical cancer cell lines [9]. This work demonstrates the possible role of formulation in enhancing the potency of paclitaxel in various cell lines. In this work, we aim to improve the potency of paclitaxel by prodrug synthesis, nanoparticle formulation, and formulation with lapatinib for synergistic effects for potential ovarian cancer treatment.

As an alternative to direct self-assembly of an amphiphilic prodrug, encapsulation of hydrophobic prodrug via nanoprecipitation methods has been a versatile approach that has also been examined. For example, vitamin E-based prodrugs have been especially promising for achieving nanoparticle formulations with high prodrug loading capacity and improved stability [10]. Zhao and Feng formulated paclitaxel in vitamin E-polyethylene glycol (PEG) nanoparticles and examined their use in breast cancer [11]. Another study synthesized silicate ester derivatives of paclitaxel with a range of hydrolysis rates and hydrophobicities. The drug release was found to be pH dependent i.e., faster at lower pH. However, the mechanism of release was not fully discussed. Additionally, the potency of the prodrugs in vitro decreased as indicated by the increase in IC_50_ concentration compared to paclitaxel in breast cancer MDA-MB-231 cells [12]. In other work, Ansell et al. formulated a series of hydrophobic paclitaxel prodrugs with various lipids including α-tocopherol (vitamin E) and were conjugated via diglycolate or succinate linkages [13]. The prodrugs were encapsulated into polymer nanoparticles via Flash NanoPrecipitation. In this case, the potency of the prodrugs in vitro decreased as indicated by the increase in IC_50_ concentration compared to paclitaxel in various cell lines, including A2780, an ovarian cancer cell line. Furthermore, drug release from the nanoparticle was not reported. These studies have established that formulating hydrophobic paclitaxel prodrugs into nanoparticles is possible but can affect their potency in vitro. This decrease in potency may be related to the release and subsequent hydrolysis of the prodrug. However, the effect of prodrug synthesis on drug release has not been fully elucidated.

In this study, we use Flash NanoPrecipitation of tannic acid and iron as a pH-labile nanoparticle platform for enhancing paclitaxel potency. Formulation of tannic acid iron nanoparticles incorporating weakly hydrophobic drugs such as paclitaxel has recently been reported [14]. Building on this work, our aim is to improve the potency of paclitaxel with a combination of prodrug synthesis, nanoparticle formation, and formulation with lapatinib with synergistic effects. Therefore, we conjugated paclitaxel to vitamin E to produce a hydrophobic paclitaxel prodrug and evaluated its potency in vitro using ovarian cancer cell line OVCA-432 as a model system. The prodrug was incorporated into nanoparticles and the drug release kinetic was studied. Specifically, we studied drug release mechanism of the prodrug compared to paclitaxel. The effect of formulation on drug potency using IC_50_ as a measure of potency was tested. Finally, potential synergistic effects when the prodrug was co-encapsulated with other chemotherapeutic agents, specifically lapatinib, were examined.

## 2. Results and Discussion

### 2.1. Prodrug Formulation with Enhanced Potency In Vitro

Modifying paclitaxel to produce hydrophobic prodrugs is a well-established method for enabling nanoparticle formulation [5,6]. In this work, we examine the potency of a hydrophobic paclitaxel prodrug formulated into pH-labile nanoparticles using Flash NanoPrecipitation, a rapid, scalable approach. The first step was synthesis of the prodrug.

**Figure 1:**
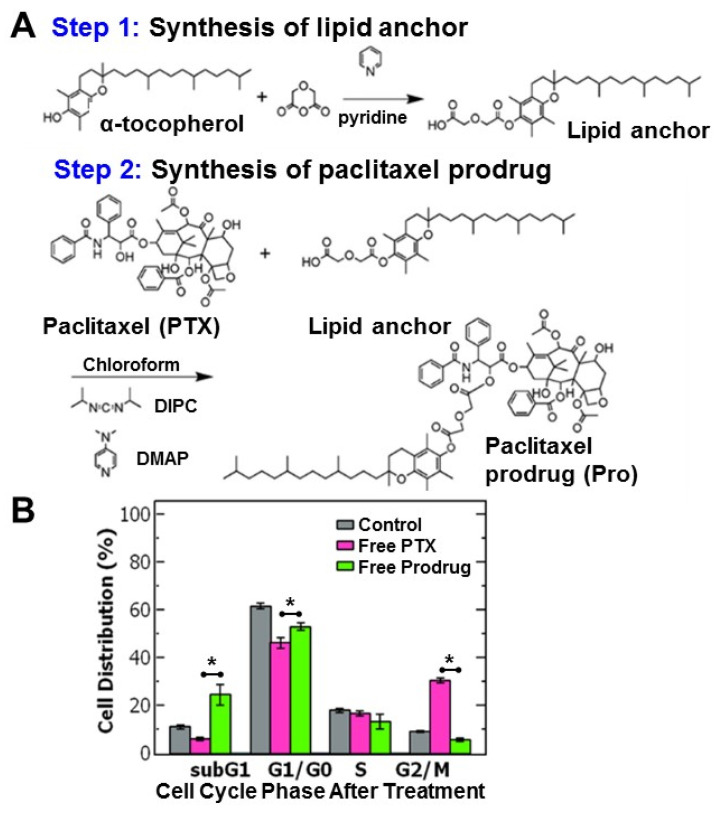
(**A**) Schematic of the two-step synthesis of the paclitaxel-prodrug with α-tocopherol as the lipid anchor. In the first step, the tocopherol lipid anchor is synthesized, and in the second step paclitaxel (PTX) is conjugated to the lipid anchor to form the prodrug (Pro). (**B**) Cell cycle analysis of ovarian cancer cell line OVCA-432 using flow cytometry of (grey) untreated cells, control, and cells treated with either (pink) free paclitaxel or (green) free paclitaxel prodrug (*n* = 3). Analysis with a t-test found a significant difference between free paclitaxel and free prodrug was when comparing the subG_1_ phase (*p* = 0.018), G_1_/G_0_ phase (*p* = 0.011), G_2_/M phase (*p* = 0.00005) where the cell cycle is comprised of the: G_1_ = growth phase 1, S = DNA synthesis phase, G_2_ = growth phase 2, M = mitosis, and G_0_ = rest phase.

The hydrophobic paclitaxel prodrug (Pro) was produced via conjugation to α-tocopherol (Vitamin E) based on previous reports (Figure 1A) [13]. The paclitaxel-prodrug was synthesized by a two-step reaction using a diglycolic anhydride linker. In the first step of the reaction the lipid anchor α-tocopherol reacted with diglycolic anhydride in pyridine to form a carboxylic acid group on the lipid anchor. The product was extracted and conjugated to paclitaxel via an esterification reaction with paclitaxel using diisopropylcarbodiimide. This reaction forms a covalent link between the hydroxyl group on the paclitaxel (at the 2′-OH position) and the carboxylic acid group on the lipid anchor forming an ester bond [13]. The reaction yield was ~60%. Analysis of the product by direct-infusion electrospray mass spectrometry is consistent with the expected prodrug (Figure S1). The reaction product was also characterized using ^1^H-NMR (Figure S2). The ^1^H-NMR spectrum was comparable to data previously reported by Ansell et al. [13]. Combined these analyses confirm successful formation of the expected paclitaxel prodrug. Recovery of the paclitaxel expected by hydrolysis of the C2′ ester [6,13,15].

The potency of the hydrophobic paclitaxel prodrug was compared to paclitaxel following synthesis. The dose response in vitro was measured with ovarian cancer cells (OVCA-432) as a model system. The cells were treated with either paclitaxel or prodrug with a range of concentrations. Measuring cell viability, the half-maximal inhibitory concentration (IC_50_) was used as a measure of potency. A lower IC_50_ indicates higher drug potency. Treating the ovarian cancer cells with free prodrug resulted in an IC_50_ of 10 ± 5 μM which was over 8-fold lower compared to free paclitaxel (83 ± 6 μM) (Table S1).

These results suggest that formulating hydrophobic prodrug of paclitaxel is an approach for increasing the potency of paclitaxel to treat ovarian cancer cells. The increase in potency is surprising as hydrophobic paclitaxel prodrugs have generally had higher IC_50_ values than free paclitaxel by 20 to 70-fold [12]. Interestingly, the result is comparable to paclitaxel conjugated to a poly(ethylene glycol)-b-poly(acrylic acid) block copolymer, which was pH degradable [6].

To better understand the efficacy of paclitaxel and the hydrophobic prodrug on the ovarian cancer cells, we examined the cell cycle distribution (where the cell cycle is comprised of the: G_1_ = growth phase 1, S = DNA synthesis phase, G_2_ = growth phase 2, M = mitosis and G_0_ = rest phase). Paclitaxel is known to arrest cells in the G_2_/M phase by stabilizing microtubules and preventing their disassembly necessary for cell division [16].

Cells were treated with paclitaxel or the paclitaxel prodrug at the IC_50_ concentration and then analyzed using flow cytometry. Untreated OVCA-432 cells were primarily distributed in the G_1_/G_0_ phase at 62 ± 1.3% and only 9 ± 0.3% of cells were in the G_2_/M phase. Treating the cells with free paclitaxel reduced the percentage of cells in the G_1_/G_0_ phase to ~46% and stabilized the cells in the G_2_/M phase (~30%). Interestingly, while the prodrug also reduced the percentage of cells in the G_2_/M phase, there was greater accumulation of cells in the subG_1_ phase (~25%) compared to the control and free paclitaxel (*p* = 0.018) rather than the G_2_/M phase (~6%) (Figure 1B). The greater accumulation of cells in the subG_1_ phase treated with the prodrug could indicate cells spend less time in the G_2_/M phase and transition to the subG_1_ phase due to cell damage. Increased proportion in the subG_1_ phase has been attributed to shorter arrest in the G_2_/M phase with rapid DNA fragmentation leading to cell death overtime [17,18]. Overall, these results are comparable to previous reports [14] and support the observed increase in potency of the prodrug compared to paclitaxel.

### 2.2. Formulation of Prodrug Nanoparticles

To further increase the potency, we next examined formulating the prodrug into nanoparticles since encapsulation of hydrophobic drugs into nanoparticles can increase their potency [9,14,19,20]. Thus, we used Flash NanoPrecipitation (FNP), a rapid, scalable, polymer directed self-assembly method [8,21]. Building on our previous work, we incorporated the prodrug into a pH-labile nanoparticle based on a tannic acid (TA) coordination complex with iron [14,22]. Our goal was to apply the fundamental understanding of the self-assembly process to achieve prodrug loaded nanoparticles that were less than 200 nm in diameter and uniform by dynamic light scattering (DLS) (that could enable passive targeting [23,24]). Since the formulation of paclitaxel was recently reported, in this study, we focus on examining drug release and evaluating potency of the formulations. Therefore, we formulated each the prodrug to maximize drug loading while achieving uniform particles (by DLS) that were less than 200 nm for comparison to the previously reported PTX formulation with maximal drug loading.

Briefly, nanoparticles were prepared by dissolving the amphiphilic block co-polymer, polystyrene-b-polyethylene glycol (PS-b-PEG) stabilizer, tannic acid (TA), and the paclitaxel prodrug (Pro) in a water miscible organic solvent (e.g., tetrahydrofuran, THF). This organic stream was rapidly mixed with Fe^3+^ (aq.) in a confined impinging jet (CIJ) mixer. Upon mixing, an insoluble tannic acid-iron (TA-Fe) complex formed. Simultaneously, precipitation of the prodrug and self-assembly of the PS-b-PEG occurred. The formation of the TA-Fe complex facilitated incorporation of the precipitating drugs. Ultimately, the growth of the nanoparticle core was kinetically stabilized by adsorption of the block co-polymer onto the surface of the core.

With this FNP nanoparticle platform involving tannic acid and iron coordination complexation, the two formulation parameters that primarily affect nanoparticle size and polydispersity are the drug concentration and the ratio of the block co-polymer to core (BCP: core) [21,22,25]. As a starting point we used the formulation parameters that resulted in ~100 nm PTX-loaded nanoparticles, uniform by DLS with the highest drug loading. Specifically, we formulated prodrug loaded nanoparticles using a prodrug concentration at 1 mg/mL in the organic stream at a constant 2:1 ratio of the BCP:core as previously described for paclitaxel loaded nanoparticles (PTX NPs) [14]. However, the resulting dispersion was not uniform by DLS with a peak at 184 ± 11 nm and a secondary peak at 26 ± 2 nm. The nanoparticles on the order of ~30 nm can be attributed to empty block copolymer micelles [14,26]. When the prodrug concentration was reduced to 0.5 mg/mL the primarily particle size decreased to ~155 nm but also contained a secondary micelle peak. By further reducing the drug concentration to 0.25 mg/mL, monodispersed nanoparticle were produced at a size of 135 ± 6 nm and a polydispersity (PDI) of 0.206 ± 0.017 (Table S2). For the sample prepared at the intermediate prodrug concentration, the size distribution of the resulting nanoparticle dispersion (in phosphate buffered saline) was relatively stable with no significant change in peak sizes or PDI over several weeks (Table S3).

**Figure 2:**
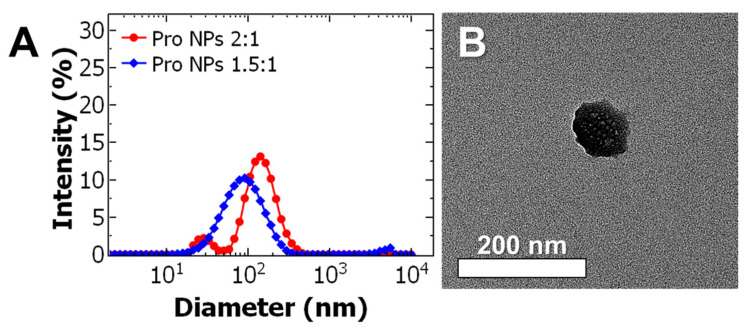
(**A**) Representative dynamic light scattering (DLS) results of prodrug nanoparticle (Pro NP) formulations. The ratio of the block co-polymer to core (BCP: core) was varied from 2:1 ratio (red) to 1.5:1 (blue) at a constant drug concentration of 0.5 mg/mL. Decreasing the BCP:core ratio from 2:1 to 1.5:1 produced uniform 98 ± 4 nm diameter particles. (**B**) Representative transmission electron microscopy (TEM) images of Pro NPs formulated at 1.5:1 ratio and 0.5 mg/mL of the paclitaxel prodrug taken at 40 kX (scale bar = 200 nm).

Uniform particles could be achieved but at relatively low nominal drug loading. To increase the nominal drug loading, the effect of the BCP: core ratio was examined at an intermediate drug concentration of 0.5 mg/mL in the organic stream. Decreasing the ratio from 2:1 to 1.5:1 resulted formation of monodispersed nanoparticles at 98 ± 4 nm with a PDI of 0.233 ± 0.008 (Figure 2A). TEM analysis confirms the particles are spherical and the particle size is consistent with DLS (Figure 2B, with additional TEM images shown in Figure S3).

These results demonstrate that at a desired drug concentration there is a narrow window of block copolymer to core material ratios that result in formation of uniform particles, which is consistent with previous work [14,22]. Self-assembly of uniform particles requires appropriate matching of timescales between nucleation of the core (TA-iron complexation and precipitation of the prodrug) and self-assembly of the block co-polymer. By matching the timescales, we minimized the formation of empty micelles and allowed for higher nominal drug loading. This principle has been previously described and studied for other hydrophobic materials encapsulated via FNP [8,21,25,26].

### 2.3. Nanoparticle Encapsulation Efficiency and Drug Loading

Following nanoparticle formulation, nanoparticle mass in dispersion was determined by thermogravimetric analysis (TGA) and the drug concentration in the particles was determined by disassembling the nanoparticles with acetonitrile and measuring the encapsulated drug concentration by high performance liquid chromatography (HPLC) to determine encapsulation efficiency and drug loading. Encapsulation efficiency (EE%) and drug loading (DL%) were calculated based on Equations (1) and (2), respectively, and the values reported are the average and standard deviation of three trials of three separate FNP formulations:

(1) $$\mathrm{encapsulation}\;\mathrm{efficiency}\;\left(\mathrm{EE}\%\right)=\;\frac{\mathrm{Mass}\;\mathrm{of}\;\mathrm{drug}\;\mathrm{encapsulated}}{\mathrm{Initial}\;\mathrm{mass}\;\mathrm{of}\;\mathrm{drug}}\times \;100$$

(2) $$\mathrm{drug}\;\mathrm{loading}\;\left(\mathrm{DL}\%\right)=\;\frac{\mathrm{Mass}\;\mathrm{of}\;\mathrm{drug}\;\mathrm{encapsulated}}{\mathrm{Total}\;\mathrm{nanoparticle}\;\mathrm{mass}}\;\times \;100$$

The encapsulation efficiency was determined by comparing the drug mass encapsulated to the nominal amount in the formulation using Equation (1). The encapsulation efficiency was comparable between the paclitaxel loaded nanoparticles (PTX NPs) and the paclitaxel prodrug loaded nanoparticles (Pro NPs) at ~40% (Table 1). The encapsulation efficiency of the two nanoparticles suggests comparable drug affinity of the paclitaxel and the prodrug to the nanoparticle core during the FNP process. This is worth noting, as typically more hydrophobic agents (logP > 6) are observed to have greater encapsulation efficiency in nanoparticles formulated with FNP [8,27,28]. The standard deviation represents batch to batch variation and is comparable to previous reports [14]. Increasing the hydrophobicity also seemed to improve the batch to batch variability in encapsulation efficiency as indicated by the lower coefficient of variation (standard deviation/average) for prodrug (4%) compared to paclitaxel (38%) (Table 1). These results could suggest that the interaction with the TA-iron complex guides the drug encapsulation process and is comparable between paclitaxel and the prodrug at the respective concentrations.

The drug loading was determined by comparing the encapsulated drug mass to the total mass of the nanoparticle dispersion using Equation (2). Comparing the drug loading of paclitaxel and prodrug in the single-drug loaded nanoparticles there was a 2.5-fold higher drug loading of paclitaxel compared to the prodrug (Table 1). The difference in drug loading can be attributed to a half the prodrug concentration (0.5 mg/mL) compared to paclitaxel (1 mg/mL) used for the formation of uniform nanoparticles. Further work to understand the effect of hydrophobicity and initial concentration on encapsulation and drug loading would be useful to formulate nanoparticles with tunable size and drug loading. In this study, we focus on examining drug release and evaluating potency of the formulations presented in Table 1.

### 2.4. Drug Release

Encapsulating paclitaxel in pH-degradable polymer micelles increases its potency [6,9]. Similar to the acetal linkages used in the system described by Gu et al. [9], the tannic acid-iron complex core provides a pH-labile nanoparticle platform for paclitaxel prodrug release. Specifically, the TA-iron complex nanoparticle core is insoluble above pH 7 and soluble below pH 5 [22,29]. Thus, we examined the drug release from the nanoparticles under two pH conditions via dialysis: pH 7 when the nanoparticle platform is stable and as well as pH 4 when some nanoparticle disassembly may occur [30,31,32]. The nanoparticles were dialyzed against either 1X PBS (phosphate buffered saline) at pH 7 with 0.5% Tween 80 or 50 mM acetate buffer at pH 4 with 0.5% Tween 80. The nanoparticle compartment was sampled to track the disappearance of drug from the nanocarriers and the drug concentration remaining in the nanoparticles at each time point was measured with HPLC to determine the drug release profile similar to established methods [33,34]. The focus of this work was to compare the release mechanism of paclitaxel and the prodrug when the nanoparticle is stable and at a pH when some nanoparticle disassembly is expected [22].

**Figure 3:**
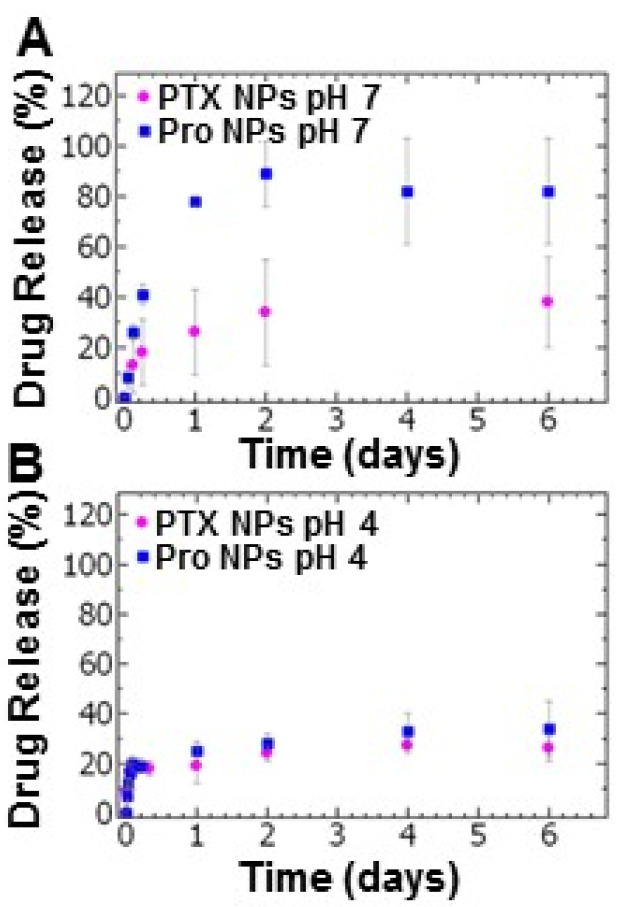
The drug release profiles of paclitaxel nanoparticles (PTX NPs) (pink) and prodrug nanoparticles (Pro NPs) (blue) at (**A**) pH 7 and (**B**) pH 4 of (pink) *(n* = 3, error bars represent standard deviation of the 3 trials).

We compared the drug release of paclitaxel and the prodrug from the tannic acid-iron nanoparticle platform. Examining the drug release at pH 7, the PTX NPs exhibited burst release over the first 6 h at which ~20% of the paclitaxel has been released. Under the same conditions, ~40% of the prodrug had been released during the first 6 h (Figure 3A). During the sustained drug release period examined for up to 6 days, the release of paclitaxel was limited with a cumulative drug release of ~40% whereas the maximum prodrug release from the Pro NPs was ~90% (Figure 3A). The relatively low cumulative release of paclitaxel may indicate an equilibrium between nanoparticle core and bulk solution (Tween 80 in PBS). These results are comparable to results reported for other micellar systems [35].

Under acidic conditions (pH 4), burst release was observed within one hour of dialysis followed by a period of slow release measured over 6 days. The total paclitaxel released within the first hour was 17 ± 3%. This result was comparable to the prodrug (16 ± 5%) (Figure 3B). The total drug release after 6 day at pH 4 for the PTX NPs and Pro NPs was ~26% and ~34%, respectively (Figure 3B). The low cumulative release of both PTX and prodrug at acidic conditions may indicate an equilibrium between partially disassembled nanoparticles and bulk solution (Tween 80 in sodium acetate buffer).

Surprisingly, more prodrug was released from the nanoparticles compared to paclitaxel despite being more hydrophobic. To further understand the drug release, we examined the mechanism of drug release. Drug release at pH 7, under which the nanoparticles are stable, was fit the data to the Korsemeyer-Peppas diffusion model (Equation (3)):

(3) $$\frac{M_{t}}{M_{\infty }}={\mathrm{at}}^{n}$$

where the *M_t_* is the drug release at time, *t*, *M_∞_* is maximum drug release, and *a* is the release rate. The diffusion exponent, *n*, is determined based on the fit and describes the drug release mechanism [36]. The Korsemeyer-Peppas model is used in cases were the nanoparticle are assumed to be stable with no change in size where the diffusion exponent is used to classify the transport mechanism e.g., Fickian diffusion, non-Fickian transport, etc.

For cumulative release below 70% [36,37] at pH 7, the Korsemeyer-Peppas model appeared to correlate reasonably well with the data with an coefficient of determination, R^2^ > 0.98. We also note that the observed rate constant (*a*) for the Pro NPs was 1.8-fold greater than PTX NPs (Table 2), suggesting that the apparent release rate of the paclitaxel prodrug from the nanoparticles is faster than paclitaxel.

The diffusion exponent for PTX NPs was less than 0.45 indicating first order Fickian diffusion [38,39] (Table 2). Fickian diffusion describes drug release when the rate of diffusion is substantially greater than the polymer chain relaxation of block co-polymer [40]. Under this condition, the release of paclitaxel driven by the concentration gradient between the nanoparticle core and bulk solution.

In contrast, the diffusion exponent for the Pro NPs was greater than 0.89 indicating Super Case II transport (Table 2). This transport describes a system in which outer layer of the nanoparticles prevents swelling of the nucleus and instead leads to compression of the nucleus and penetration of the solvent which eventually results in disassembly of the nanoparticle [40]. The rate of prodrug release is therefore not dependent on rate of diffusion but instead the compressive stresses on the nanoparticle core leading to rapid drug release. Furthermore, the unexpected rapid release of the prodrug relative to paclitaxel can be attributed to a difference in drug release mechanisms and not on the drug hydrophobicity. The compressive stresses on the nanoparticle core suggest a strong interaction between the hydrophobic block of the block co-polymer and the prodrug. Interestingly, these results suggest that for this nanoparticle platform, the mechanism of release can be tuned by varying the drug properties (e.g., molecular weight, hydrophobicity).

For the drug release at pH 4 when the TA-Fecore transitions from an insoluble to soluble form which is expected to cause some dissolution of the nanoparticle core, we found that the Korsemeyer-Peppas was a poor fit and instead used the Hixon-Crowell diffusion model (Equation (4)), which describes drug release from a dissolving core or dissolving tablet [36,41]:

(4) $${\left(1-\;\frac{M_{t}}{M_{0}}\right)}^{1/3}=1-{\;K}_{\beta }t$$

where *M*_0_ is the initial amount of drug dose therefore *M_t_*/*M*_0_ is the fraction of total drug released. *K_β_* is the release constant, which is dependent upon the change in surface and volume of the nanoparticles.

With the Hixson-Crowell model, we fit the experimental data from pH 4 conditions for PTX NPs and Pro NPs. This model provided higher R^2^ values than the Korsemeyer-Peppas model (R^2^ values using the Korsemeyer-Peppas model are provided in Table S4 for comparison) indicating better correlation with the experimental data. Interestingly, we found that the rate constants for PTX NPs and Pro NPs were equivalent during burst and sustained release phases (Table 3). 

The similarity in release rates observed with the Hixson-Crowell model indicates that the drug release kinetics are driven by core solubility and particle disassembly at pH 4. The relatively low cumulative release may indicate an equilibrium between partially disassembled nanoparticles and bulk solution.

Overall, these observed results that indicate that the pH-labile nanoparticles are a useful platform for modulating drug release kinetics. Notably, comparing the paclitaxel and prodrug at pH 7, prodrug synthesis can affect the release mechanism. Further evaluation of stability and release in more biologically relevant media such as full growth medium with serum [14,42,43] as well as biodistribution in vivo to how understand the drug release and prodrug hydrolysis are affected by protein binding [44] are of interest but are outside the scope of this study.

### 2.5. Prodrug Nanoparticle Potency and Evaluating the Cell Cycle Distribution

**Figure 4:**
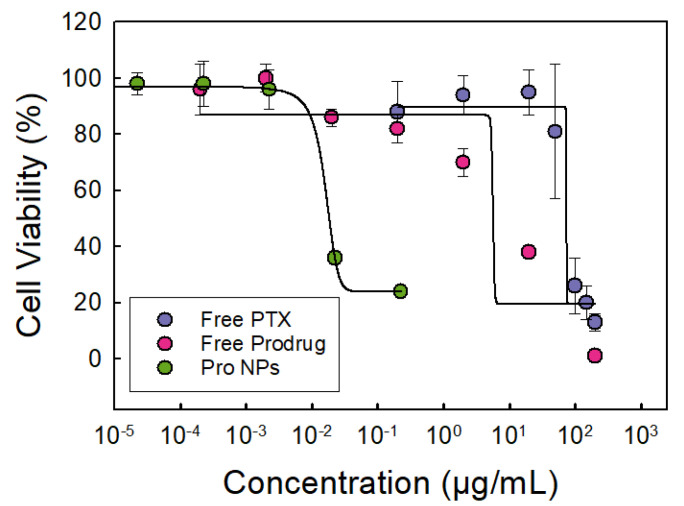
Dose–response curve of OVCA-432 cells treated with either free paclitaxel (PTX) shown in purple, free paclitaxel prodrug (Pro) shown in red, or prodrug nanoparticles (Pro NPs) shown in green. The dose–response curve of free prodrug shifted to lower drug concentrations compared to free paclitaxel indicating an increase in potency. The Pro NPs further shifted the dose–response curve to lower IC_50_ values indicating further increase in potency. Tannic acid-iron nanoparticles did not significantly affect cell viability (>90%) at the concentrations used (*n* of 6, error bars represent standard deviation of the 6 trials).

Next, the potency of the prodrug nanoparticle formulation was assessed in vitro using IC_50_ as a measure of potency with ovarian cancer cells, OVCA-432 cells as a model system. We note that we have previously established the cytocompataiblity of the TA/Fe only nanoparticle platform [14]. The viability of the cells treated with TA/Fe nanoparticles was greater than 90% for concentrations up to 100 μg/mL, i.e., three orders of magnitude higher than the concentrations presented in Figure 4.

The focus of this work was evaluating the potency of the prodrug and prodrug nanoparticle formulations. In Figure 4, we compare the dose response of cells treated PTX, prodrug or with nanoparticles loaded prodrug. The prodrug was more potent than paclitaxel; the dose response curve shifted to the left. Synthesizing the prodrug enhanced the potency as indicated by the 8-fold decrease in IC_50_ concentration (Table 4). 

Formulating the prodrug into nanoparticles further enhanced the potency of the prodrug significantly from 10 ± 5 μM to 0.009 ± 0.002 μM (Figure 4 and Table 4). The 1100-fold decrease in IC_50_ concentration indicating an increase in prodrug potency in this nanoparticle formulation is notable. The increase in potency has been observed in other polymer nanoparticle systems [19,45,46], but it is not fully understood. The significant increase in prodrug potency in the TA-Fe nanoparticles could be attributed to sustained release over the 48 h treatment period thereby increasing bioavailability [19,47,48].

Since we have previously evaluated the potency of PTX NPs [14], we also compared the performance of the prodrug loaded nanoparticles with PTX NPs. Excitingly, the Pro NPs exhibited greater potency compared to PTX NPs. Specifically, the Pro NPs were 5-fold more potent compared to the PTX NPs (per mole of paclitaxel) despite the higher drug loading in PTX NPs (Table 4). Thus, we use both prodrug synthesis and nanoparticle formulation to enhance the potency of paclitaxel using OVCA-432 as a model cell line.

Interestingly, these results differ from previous studies investigating the formulations of hydrophobic paclitaxel prodrugs [12,13,49]. Ansell et al., observed a 10-fold decrease in potency in ovarian cancer cells (A2780) and a 3-fold decrease in potency in breast tumor cells (MCF-7) when comparing α-tocopherol conjugated paclitaxel prodrug to paclitaxel that were co-encapsulated with phosphatidylcholine (POPC) in polymer nanoparticles stabilized by PS-b-PEG via FNP [13]. The difference in the observed results could indicate cell-dependent cytotoxicity due to the gene expression [50]. The difference in nanoparticle formulation methods, co-encapsulation with TA-Fe versus a non-labile nanoparticle platform; may also play a role as the pH labile platform may increase the bioavailability of the prodrug [51]. Additionally, the diameter of the nanoparticles formulated with TA-Fe were ~100 nm which are 4-fold larger compared to those formulated by Ansell et al. (~25 nm). Nanoparticle size plays a significant role in the mechanisms of endocytosis [52,53]. Overall, our results indicate that prodrug synthesis and nanoparticle formulation can enhance the potency of paclitaxel and may be useful treating ovarian cancer.

**Figure 5:**
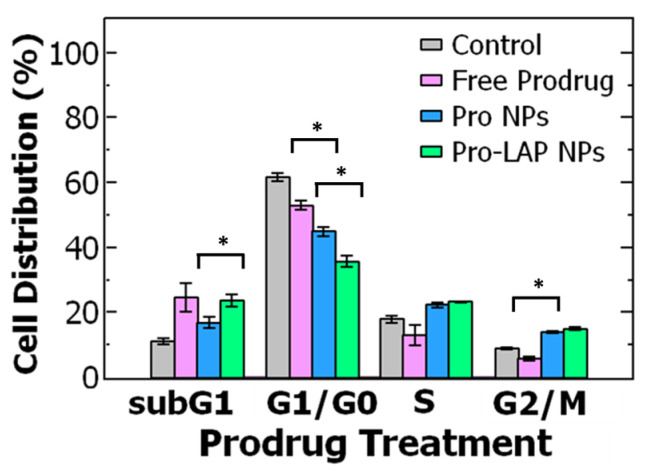
Cell cycle analysis of ovarian cancer cell line OVCA-432 using flow cytometry comparing (grey) untreated cells, the control, to three different paclitaxel prodrug (Pro) formulations. The cell cycle is comprised of the: G_1_ = growth phase 1, S = DNA synthesis phase, G_2_ = growth phase 2, M = mitosis, and G_0_ = rest phase. The cell cycle distribution was examined for free prodrug, single-drug loaded paclitaxel nanoparticles (Pro NPs), and co-loaded nanoparticle encapsulation prodrug and lapatinib (Pro-LAP NPs). The graph shows the average ± standard deviation with an *n* of 3. Comparing cells treated with free prodrug and Pro NPs with a *t*-test found a significant difference in G_1_/G_0_ phase (*p* = 0.0002) and G_2_/M phase (*p* = 0.00003). A *t*-test comparing Pro NPs and Pro-LAP NPs found a significant difference in G_1_/G_0_ phase (*p* = 0.001) and subG_1_ phase (*p* = 0.012).

To further probe the increase in potency upon encapsulation, we examined the cell cycle distribution when the OVCA-432 cells were treated with free prodrug and Pro NPs. As previously described, the untreated cells primarily accumulated in the G_1_/G_0_ phase. When the cells are treated with Pro NPs, there was a significant decrease in cells in the G_1_/G_0_ phase (*p* = 0.0002) than when treated with free prodrug (at their respective IC_50_ concentrations). The cells treated with Pro NPs redistributed to the G_2_/M phase (~14%) and subG_1_ phase (~17%) (Figure 5). When treating with the Pro NPs there was a greater percentage of cells in the G_2_/M phase and lower percentage of cells in the subG_1_ phase compared to the free prodrug. These results suggest that encapsulating the paclitaxel prodrug into nanoparticles enhances G_2_/M arrest and accumulation in the subG_1_ phase leading to DNA fragmentation and cell death [17,18]. Future studies to investigate intracellular paclitaxel drug accumulation and complementary biological assays would be interesting.

### 2.6. Synergy of Drug Combination with the Paclitaxel Prodrug

Finally, we examined if the prodrug could be formulated with other chemotherapeutic agents with synergistic effects building our previous work and previous reports [14,19,54,55]. Lapatinib iscommonly paired with paclitaxel to overcome drug-resistant mechanisms. Specifically, lapatinib is a tyrosine kinase inhibitor that inhibits P-glycoprotein (P-gp), which pumps out paclitaxel and lowers the intracellular drug accumulation. P-gp is often overexpressed in ovarian cancer. Therefore, lapatinib was selected for examining the efficacy of combination drug delivery with the hydrophobic paclitaxel prodrug [56,57].

**Figure 6:**
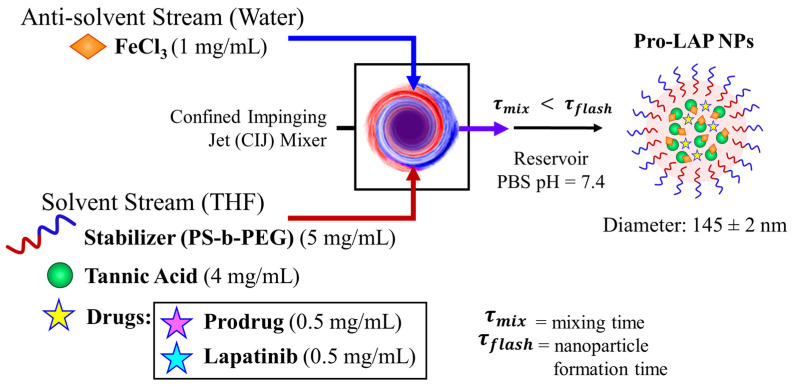
Schematic of paclitaxel prodrug (Pro) and lapatinib (LAP) co-loaded nanoparticle formulation with Flash NanoPrecipitation (FNP). The drugs are encapsulated by in situ tannic acid (TA) and iron coordination complex using an amphiphilic block copolymer stabilizer, polystyrene-b-polyethylene glycol (PS-b-PEG). The organic stream containing PS-b-PEG, TA, prodrug, and lapatinib, is rapidly mixed with Fe^3+^ in a confined impinging jet (CIJ) mixer. The resulting nanostructures are 145 ± 2 nm in diameter.

We formulated nanoparticles co-encapsulating paclitaxel prodrug and lapatinib. To achieve uniform particles, the total drug concentration was maintained at 1 mg/mL with equal concentrations of the two drugs (0.5 mg/mL of prodrug and 0.5 mg/mL of lapatinib), as previously observed for paclitaxel and lapatinib combinations [14] and a block copolymer to core ratio of 1:1 was selected (Figure 6 with TEM images in Figure S4). The size of the particles was comparable to the other formulations; the average diameter by dynamic light scattering was 145 ± 2 nm with a PDI of 0.111 ± 0.018 (Table S5). The drug loading of prodrug and lapatinib loaded nanoparticles (Pro-LAP NPs) was 2.11 ± 0.50 wt.% and 0.79 ± 0.40 wt.%, respectively (Table S6).

Next, we examined the potency of the co-loaded nanoparticles relative to those only loaded with the prodrug. There was a 2-fold decrease in the IC_50_ of the prodrug when it was co-encapsulated with lapatinib (from ~0.009 to 0.004 μM) compared to the prodrug nanoparticles (Table 4). Therefore, including lapatinib in the formulation further increases the potency of the paclitaxel in the formulation. Furthermore, the synergy of the drug combination was examined by determining the combination index (CI), given by Equation (5):

(5) $$\mathrm{CI}=\frac{{\mathrm{IC}}_{50}{\left(A\right)}_{\mathrm{pair}}}{{\mathrm{IC}}_{50}\left(A\right)}+\frac{{\mathrm{IC}}_{50}{\left(B\right)}_{\mathrm{pair}}}{{\mathrm{IC}}_{50}\left(B\right)}$$

where the IC_50_ concentration for drug A in combination (*IC_50_(A)_pair_*) is divided by the IC_50_ of drug A alone (*IC_50_(A)*) and added to that of drug B. A CI less than 1 indicates synergistic drug interaction while a CI equal to or above 1 indicate additive or antagonistic interaction, respectively. We determined that the co-loaded Pro-LAP NPs had a CI of 0.51. The result indicates that formulation promotes synergism between the prodrug and lapatinib.

We examined the drug release from the co-loaded nanoparticles at pH 7 and pH 4 with 0.5% Tween 80. Based on the results from the PTX NPs and prodrug loaded nanoparticles at pH 7 and pH 4, the release data at pH 7 was fit using the Korsemeyer-Peppas model and the data at pH 4 was fit using the Hixon-Crowell diffusion model to confirm the mechanism of release. At pH 7, the diffusion exponent release and rate constant of the prodrug from the co-loaded nanoparticle were similar to the prodrug released from the prodrug loaded nanoparticle. Further, the diffusion exponent for the lapatinib was greater than 0.89 (Table S7) suggesting Super Case II transport in which release of both drugs occurs due to compressive stresses on the nanoparticle core rather than diffusion. Lapatinib had a higher rate constant, but lower cumulative release. Examining LAP release, we observe a decrease in cumulative release after 24 h at pH 7 (Figure S5) similar to previous reports which has been attributed to supersaturation and nanopreciptaiton of lapatinib in the dialysis media [14,58,59].

Examining release of the prodrug from the co-loaded nanoparticles at pH 4, higher cumulative release of the prodrug was observed than the prodrug released from prodrug only-loaded nanoparticles. This result suggests that the pH may affect the prodrug equilibrium concentration between partially disassembled nanoparticles and bulk solution, which affects the drug release kinetics. The rate constants from the Hixson-Crowell diffusion model were significantly higher than from the co-loaded nanoparticle than the prodrug only nanoparticle for both burst and sustained release (Table S8). At pH 4, lapatinib had lower release rate constants than the prodrug during burst and sustained release resulting in lower sustained release (cumulative release ~50% for prodrug compared to ~30% for lapatinib after 6 days). This low cumulative release may indicate an equilibrium between partially disassembled nanoparticles and bulk solution, which has been observed in other micelle systems. Additionally, we observe a decrease in cumulative release after 3 h at pH 4 (Figure S6) similar to previous reports which has been attributed to supersaturation and nanopreciptaiton of drugs in the dialysis media [14,58,59]. Thus, further studies of the stability and release in more biologically relevant media such as full growth medium with serum [14,42,43] as well as biodistribution in vivo to how understand the drug release and prodrug hydrolysis are affected by protein binding are of interest but are outside the scope of this study.

Taken together, these results suggest that the prodrug is released prior to lapatinib resulting in sequential drug delivery. Synergistic effects resulting from such sequential delivery of paclitaxel followed by lapatinib has been previously observed [54,60,61]. Thus, the observed synergistic interactions between the prodrug and lapatinib could, in part, be due to mechanism of prodrug release from the nanoparticle platform.

Further, we examined the effect of treatment with the co-loaded nanoparticles on cell cycle distribution. The Pro-LAP NPs exhibited the greatest reduction in the proportion of cells in the G_1_/G_0_ phase compared to both free prodrug and Pro NPs. The cells were redistributed among the other three phases. Particularly, there was a significant increase in cells in the subG_1_ phase from ~17% when treated with Pro NPs to ~24% when treated with Pro-LAP NPs (*p* = 0.012). The proportion of cells in the G_2_/M phase was equivalent between the two treatments (Figure 5). The greater portion of cells in the subG_1_ phase with Pro-LAP NPs could be attributed to the greater drug potency due to combination delivery as well as coordination between cell cycle arrest induced by the paclitaxel prodrug (G_2_/M arrest) and lapatinib (G_1_/G_0_ arrest) [16,62].

Overall, these results demonstrate several approaches to improving potency of paclitaxel: prodrug synthesis, encapsulation into nanoparticles, and formulation with other anti-cancer drugs. These methods can be combined and are promising for improving the treatment of ovarian cancer. Given the drug release profiles observed (higher cumulative release at pH 7 compared to pH 4), these nanoparticles may be well suited for incorporating into oral dosage formulations, which are of increasing interest as alternatives to paternal formulations to increase patient compliance [63]. Additionally, when further formulating these nanoparticles it is important to consider that pH responsive systems may be limited by low sensitivity. It can be combined with other types of endogenous stimuli e.g., redox, enzymes to improve selectivity of drug release in diseased tissue [64]. Applying this combined approach to other types of ovarian (e.g., endometriod, A2780) and other types of cancer e.g., breast cancer would also be of interest.

## 3. Materials and Methods

### 3.1. Materials

Tetrahydrofuran (THF, HPLC grade), dimethyl sulfoxide (DMSO, HPLC grade), dichloro-methane (DCM, HPLC grade), diglycolic anhydride (97%, Alfa Aesar, Haverhill, MA, USA), diisopropylcarbodiimide (99%, Alfa Aesar, Haverhill, MA, USA), alcohol-free chloroform, acetonitrile (HPLC grade), ethanol (ACS reagent grade), methanol (ACS reagent trade), 4-(dimethylamino)pryridine (99%), anhydrous magnesium sulfate, and acetic acid (ACS reagent grade) were purchased from Fisher Scientific (Pittsburg, PA, USA). Hydrochloric acid (37%, 12 M, ACS grade), tannic acid (TA) (ACS grade), iron (III) chloride hexahydrate (97%), α-tocopherol (vitamin E, >95.5%), pyridine (anhydrous, 99.8%), deuterated chloroform CDCl_3_, and anhydrous sodium acetate were purchased from Sigma-Aldrich (St. Louis, MO, USA). Paclitaxel (PTX, >98%) and lapatinib (LAP, >98%) were obtained from Cayman Chemical Company (Ann Arbor, MI, USA); phosphate buffered saline without calcium and magnesium was purchase from Lonza (Basel, Switzerland). Polystyrene-b-polyethylene glycol (1600-b-5000 g/mol) (PS-b-PEG) was obtained from Polymer Source (Montreal, QC, Canada) and was purified by dissolving in THF (~40 °C) and precipitating into diethyl ether then dried by vacuum for two days as previously described [65].

### 3.2. Cell Culture

As a model cell line, ovarian cancer cell line OVCA-432 (human serous adenocarcinoma) was a kind gift from Xianjun Fang from Virginia Commonwealth University (collection described elsewhere [66]). The OVCA-432 cells were cultured in RPMI-1640 media containing 2 mM L-glutamine (ATCC, Manassas, VA, USA) supplemented with 10% Fortified Bovine Calf Serum (FBS, HyClone Cosmic Calf Serum, Fisher Scientific), 100 U/mL penicillin and 100 µg/mL streptomycin (Gemini Bio-Products, West Sacramento, CA, USA). The cells were cultured at 37 °C at 5% CO_2_ and passaged once a week.

### 3.3. Prodrug Formulation

A hydrophobic paclitaxel-prodrug was synthesized by conjugation to α-tocopherol (Vitamin E) lipid anchor via a two-step reaction in which the lipid anchor is reacted with diglycolic anhydride and then linked to paclitaxel using a diisopropylcarbodiimide, previously described by Ansell et al. [13]. Briefly, tocopherol (1 equiv) and diglycolic anhydride (3 equiv) reacted in pyridine at room temperature overnight. The solvent was removed using a rotovap and the residue extracted from dilute hydrochloric acid with methylene chloride. The organic fractions were dried over anhydrous magnesium sulfate and filtered, and the solvent was removed. Conversion to the appropriate acid was monitored by TLC (until 100% conversion was achieved). The resulting lipid acid was used without further purification.

Then in the second step the lipid acid anchor was conjugated to paclitaxel via an esterification reaction in which paclitaxel (1 equiv) was dissolved with the tocopherol acid anchor (2 equiv) and 4-(dimethylamino)pryridine (3 equiv) with diisopropylcarbodiimide (1.3 equiv) in chloroform. This reaction proceeded at room temperature and was monitored by TLC until most of the paclitaxel had been consumed (typically 2−4 h). The mixture was then washed with HCl (aq.) and dried over anhydrous magnesium sulfate. The crude product was purified with a silica gel column using a hexane/ethyl acetate gradient. The purified produced was recovered by evaporating the solvent. The final product was analyzed with ^1^H-NMR (Ascend 600 MHz NMR instrument, Bruker, Billerica, MA, USA) by dissolving in CDCl_3_ and direct-infusion electrospray mass spectrometry (LTQ Orbitrap Velos, Thermo Fisher, Pittsburg, PA, USA) performed the Chemical and Proteomic Mass Spectrometry Core Facility at Virginia Commonwealth University.

### 3.4. Nanoparticle Formulation

Flash NanoPrecipitation (FNP) was used to prepare polymer-based nanoparticles encapsulating the anti-cancer drugs via tannic acid-iron in situ complexation with a hand-operated confined impinging jet (CIJ) mixer, as previously described [14,22]. Briefly, PS-b-PEG (4.25–10 mg/mL), TA (4 mg/mL), and the drugs of interest (0.25–1 mg/mL) were dissolved in THF by sonicating (~40 °C) for 10 min to formulate the organic stream. The nanoparticles were either loaded with paclitaxel (PTX NPs), prodrug (Pro NPs), or prodrug with lapatinib (Pro-LAP NPs). The organic stream was rapidly mixed with the Fe^3+^ (aq., 1 mg/mL) at equal volumes (1 mL of each stream) in the CIJ mixer. The effluent from the mixer was immediately diluted in 1X PBS at pH 7.4 for a final organic solvent/water ratio of 1:9 by volume.

Within 24 h of formulation, the nanoparticles were filtered to remove the organic solvent, unencapsulated drug(s), and excess TA and iron with Amicon Ultra-2 Centrifugal filters (Amicon Ultra centrifuge filter (Ultracel 50K, 50,000 NMWL), Merck Millipore Ltd., Burlington, MA, USA) by centrifuging at 3700 rpm for ~15–30 min (5804 R 15 amp version, Eppendorf, Hamburg, Germany). The nanoparticle pellet was resuspended with 1X PBS to a nominal concentration ~25 mg/mL of total solids and stored at ~4 °C. The nanoparticles were used within 5 days of FNP to ensure there was minimal change in particle size and drug loss.

### 3.5. Nanoparticle Characterization

The size and polydispersity (PDI) of the nanoparticles were characterized after FNP using dynamic light scattering (Malvern Zetasizer ZS, Malvern Instruments Ltd., Malvern, UK). The nanoparticle size, peak 1 and peak 2 mean intensity, and PDI were measured by averaging 4 measurements at a scattering angle of 173°. The average and standard deviation of three replicate FNP samples are reported.

The nanoparticles were analyzed with transmission electron microscopy (TEM) following filtration and resuspension. Samples were prepared by diluting the filtered nanoparticle dispersions with deionized water to 1:20 by volume ratio and pipetting 5 μL three times onto a TEM grid with Formvar/Carbon support films (200 mesh, Cu, Ted Pella, Inc., Redding, CA, USA). This dilution was necessary to prevent aggregation during drying. The samples were dried under ambient conditions. Then, the samples were imaged with a JEM-1230 system (JEOL, Peabody, MA, USA) at 120 kV.

The encapsulation efficiency (EE%) and drug loading (DL%) were determined for the filtered nanoparticles. The solids concentration of the nanoparticle dispersion was determined by thermogravimetric analysis (TGA) (Pyris 1 TGA, Perkin Elmer, Waltham, MA, USA). The nanoparticle dispersion was loaded at 10 µL and the temperature was ramped up from 28 °C to 110 °C at 10 °C/min and held for 30 min at 110 °C. The final nanoparticle mass was used to determine the nanoparticle drug loading.

To determine the drug content of the nanoparticles, acetonitrile (360 µL) was added to nanoparticles (10 μL) and the sample was vortexed so that the nanoparticles would disassemble. The sample was centrifuged at 10,000× rpm for 7 min, and then the supernatant was collected for reverse-phase high performance liquid chromatography (RP-HPLC) (1260 HPLC with Quaternary Pump and UV-Vis Diode Array Detector, Agilent, Santa Clara, CA, USA) fitted with a Luna^®^ 5 µm C18 100 Å, LC Column 250 × 4.6 mm (Phenomenex, Torrance, CA, USA). Nanoparticles samples loaded with paclitaxel (PTX) were eluted with a gradient of degassed water and acetonitrile at a flow rate of 1 mL/min (0–1 min at 80:20, 1–6 of ramp up to 0:100, 6–8 min at 0:100, and ramp down to 80:20 between 8–9 min). PTX was measured at a wavelength of 228 nm with a retention time of ~8 min. The Pro NPs and Pro-LAP NPs samples were eluted with a gradient of degassed 10 mM sodium acetate buffer (pH 5.6) and methanol at a flow rate of 1 mL/min (0–5 min at 70:30, 5–16 min of ramp up to 0:100, 16–17 min of at 0:100, and 17–21 min of ramp down to 70:30). The paclitaxel prodrug was measured at a wavelength of 228 nm with a retention time of ~16 min and LAP was measured at 332 nm with a retention time of ~9 min. The concentration of each drug was determined by comparing the peak areas with the standard calibration curve.

### 3.6. In Vitro Nanoparticle Drug Release

The drug release from the nanoparticles was measured under neutral (pH 7) and acidic conditions (pH 4) to model conditions of the bloodstream and endocytosis, respectively. The drug release was measured in vitro via dialysis method using 1X PBS at pH 7 with 0.5% Tween 80 to model neutral conditions and 50 mM acetate buffer at pH 4 with 0.5% Tween 80 to model acidic conditions. Tween 80 was added to the dialysis medium to improve the solubility of the hydrophobic drugs [58].

The nanoparticle samples were prepared by first concentrating the samples using Amicon Ultra-2 Centrifugal filters (Ultracel 50K), as previously described [14]. Then, the nanoparticle samples were redispersed in either 1X PBS at pH 7 or 50 mM acetate buffer at pH 4 to a nominal total drug concentration of 1000 µg/mL. The nanoparticle dispersion (500 μL) was immediately loaded into 7000 MWCO dialysis unit (Slide-A-Lyzer^®^ MINI Dialysis Unit, Thermo Scientific, Waltham, MA, USA) and incubated with the correlating dialysis media (25 mL) at 37 °C. The dialysis media was replaced every day of the experiment. To track the disappearance of drug from the nanocarriers, the nanoparticle samples were sampled (32 μL) directly from the dialysis unit, as previously described [14,33,34] and the remaining volume was noted. Samples incubated in neutral conditions were sampled at 0 h, 1 h, 3 h, 6 h, 24 h, 48 h, day 4, day 6, and day 10 and samples incubated in acidic conditions were sampled at 0 h, 10 min, 30 min, 1 h, 2 h, 5 h, 8 h, 24 h, 48 h, day 4, day 6. The drug concentration remaining in the nanoparticles at each time point was determined by RP-HPLC as described for measuring encapsulation efficiency and drug loading. Three replicates of separate FNP nanoparticle formulations were tested for each type of nanoparticle.

The drug release from the dialysis experiments was determined by calculating the cumulative drug release at each time point based on the remaining encapsulated drug concentration determined from the HPLC data since the samples were taken from the dialysis unit. Then the samples dialyzed under neutral conditions were fit to the Korsemeyer-Peppas diffusion model (Equation (3)) by determining the fraction of the cumulative drug released at each time point relative to the maximum drug release at day 6. Then we fit a linear trend to log of the cumulative drug release relative to the log time, excluding cumulative drug release >70%. The diffusion exponent, *n*, was determined from the slope and the release constant, *a*, was determined from the intercept. Samples dialyzed under acidic conditions were fit to the Hixson-Crowell model (Equation (4)). The data was plotted as the cube root of the remaining drug versus time and the slope was determined from the linear fit as the release constant, *K_β_*. Data between *T* = 0 and 2 h was determined as the period of burst release and time point between 2 h and days were determined as the period of sustained release and was fit separately [36,41].

### 3.7. Cell Viability and Half Maximal Inhibitory Concentration

The cell viability for cells treated with free drug and nanoparticle formulations were examined for the OVCA-432 cells to determine drug potency, as previously described [14]. Briefly, cells were seeded at a density of 15 x 10^3^ cell/well in a 96-well plate with 100 µL of complete medium and incubated at 37 °C in 5% CO_2_ overnight. The media was replaced with 100 µL medium containing free-drug with 2% *v*/*v* DMSO or nanoparticles and treated for 48 h. The nanoparticles were concentrated with Amicon filters (50kDa MWCO) as described in the drug release section and the nanoparticle pellet was diluted with 1X PBS. Serial dilutions of the free drug and nanoparticle dispersion were prepared for a final drug concentration between 200–0.000002 µg/mL. Control cells were treated with complete media and an additional group of cells were treated with 2% DMSO to normalize the results from free drug treated cells. After 48 h drug treatment, the cell viability was measured with WST-1 assay (Sigma-Aldrich) according to manufacturing instructions. Briefly, the media was replaced with 100 µL of RPMI-1640 with Phenol Red (Fisher Scientific) containing 10% WST-1 and incubated at 37 °C. The samples were measured with a microplate reader (VersaMax ELISA microplate reader, Molecular Devices, San Jose, CA, USA) at a wavelength of 440 nm with background subtraction of 640 nm. The relative cell viability was expressed as a percentage of the untreated cells with mean ± standard deviation of six replicates. For these results, the half-maximal inhibitor concentration (IC_50_) of the free drug and nanoparticle formulation was determined using SigmaPlot software (Systat Software Inc., San Jose, CA, USA) from an *n* of 6.

### 3.8. Cell Cycle Analysis by Flow Cytometry

The OVCA-432 cells were seeded at a density of 20 × 10^4^ cells/mL in a 35 mm petri dish containing 3 mL of complete media. The cells were incubated at 37 °C and 5% CO_2_ until 90% confluence with the media replaced every 2 days. The cells were treated with either free drug or nanoparticle formulations for 48 h at the IC_50_ concentration of each formulation. Then, the cells were stained with Propidium Iodide (PI Flow Cytometry Kit, Abcam, Cambridge, MA, USA) for flow cytometry according to manufacturing instructions. Briefly, the cells were trypsinized and the aspirated medium and PBS were collected to minimize cell loss. The cells were centrifuged at 500× *g* for 6 min as necessary. The cells were washed with 1X PBS and fixed with 66% ethanol by slowly adding ethanol to PBS during vortexing. The cells were stored in ethanol at 4 °C for up to 4 days and then washed with PBS to remove the ethanol. The 1X Propidium Iodide and RNase solution was prepared immediately prior to use by mixing 5% *v*/*v* of 20X Propidium Iodide and 0.05% *v*/*v* 200X RNase in 1X PBS. Then the cells were resuspended in approximately 200 µL/500,000 cells of 1× propidium iodide and RNase solution and incubated in the dark at 37 °C for 30 min. Prior to flow cytometry, the cell samples filtered through a cell strainer (Falcon Test Tube with Snap Cap, Fisher Scientific). Flow cytometry was performed on a BD FACSCanto™ II Analyzer (BD Biosciences, San Diego, CA, USA) and 10,000 cells were analyzed at an excitation of 488 nm and emission of 670 nm. The samples were analyzed in triplicate.

## 4. Conclusions

In this work, we enhanced the potency of paclitaxel by conjugating it α-tocopherol (Vitamin E) to produce a hydrophobic prodrug. The prodrug is more potent than paclitaxel as indicated by the 8-fold decrease in IC_50_ concentration measured in ovarian cancer cell line, OVCA-432, used as a model system. The potency of the paclitaxel prodrug was further enhanced by incorporating into pH-labile nanoparticles. Impressively, formulating the prodrug into nanoparticles increased drug potency by 1100-fold. We also note a 5-fold increase in potency compared to PTX NPs. The cytotoxic effects were further enhanced by formulating the prodrug with lapatinib, which resulted in synergistic drug interactions as indicated by the combination index of 0.51. Overall, these results demonstrate this prodrug synthesis, nanoparticle formation, and formulation with lapatinib resulting in synergistic effects is a promising approach for enhancing paclitaxel potency. Given the drug release profiles observed (higher cumulative release at pH 7 compared to pH 4), these nanoparticles may be well suited for incorporating into oral dosage formulations, which are of increasing interest as alternatives to paternal formulations to increase patient compliance. Based on this work demonstrating that this is a promising platform for enhancing paclitaxel potency, future formulation studies to achieve nanoparticles with tunable drug loadings/ratios are of interest. Further studies to characterize the stability and drug release in biologically relevant media such as full growth medium with serum or simulated gastric fluid as well as in vivo biodistribution studies to understand how the drug release and prodrug hydrolysis are affected by protein binding would also be needed. Applying this approach to other types of ovarian (e.g., endometriod, A2780) or breast cancer would also be of interest.

## Figures and Tables

**Table 1:** Summary of the encapsulation efficiency (EE%) and drug loading (DL%) (*n* = 3)). Paclitaxel loaded nanoparticles (PTX NPs) were compared to paclitaxel prodrug loaded nanoparticle (Pro NPs).

**Samples**	**Encapsulation efficiency (EE%)**	**Drug loading (DL%)**
	**PTX/Prodrug**	**PTX/Prodrug**
PTX NPs	37.6 ± 14.4	3.11 ± 1.88
Pro NPs	45.3 ± 1.8	1.25 ± 0.22
**Samples**	**Encapsulation efficiency (EE%)**	**Drug loading (DL%)**
PTX NPs	37.6 ± 14.4	3.11 ± 1.88
Pro NPs	45.3 ± 1.8	1.25 ± 0.22

**Table 2:** Diffusion exponent (n), rate constant (a) and the coefficient of determination (R^2^) of nanoparticle drug release at pH 7 fit to the Korsemeyer-Peppas diffusion model. Paclitaxel loaded nanoparticles (PTX NPs) were compared to paclitaxel prodrug loaded nanoparticle (Pro NPs).

Sample	Diffusion Exponent (*n*)	Rate Constant (*a*)	R^2^
PTX NPs	0.3	0.7	0.99
Pro NPs	0.9	1.3	0.98

**Table 3:** Rate constant (K_s_) and coefficient of determination (R^2^) of nanoparticle drug release at pH 4 it to the Hixson-Crowell diffusion model. Paclitaxel loaded nanoparticles (PTX NPs) were compared to paclitaxel prodrug loaded nanoparticle (Pro NPs).

Sample	Burst Release		Sustained Release	
	Rate Constant (K_S_)	R^2^	Rate Constant (K_S_)	R^2^
PTX NPs	1.2	0.89	0.0072	0.82
Pro NPs	1.2	0.89	0.012	0.91

**Table 4:** The half maximal inhibitory concentration (IC_50_) of free paclitaxel (PTX), free paclitaxel prodrug (Pro), prodrug nanoparticles (Pro NPs), and prodrug-lapatinib nanoparticles Pro-LAP NPs compared to paclitaxel-loaded nanoparticles (PTX NPs) in ovarian cancer cell line OVCA-432.

Drug Treatment		IC_50_ (μM)
Free PTX		83 ± 6
Free Prodrug		10 ± 5
PTX NPs		0.047 ± 0.004
Pro NPs		0.009 ± 0.002
Pro-LAP NPs:	Prodrug	0.00442 ± 0.00001
	LAP	0.00740 ± 0.00002

## Supplementary Materials

Supplementary materials can be found at https://www.mdpi.com/1422-0067/21/23/9292/s1.
