# Supplementary material for: Self-Assembly of pH-Labile Polymer Nanoparticles for Paclitaxel Prodrug Delivery: Formulation, Characterization, and Evaluation
Source: Int J Mol Sci. 2020 Dec 5;21(23):9292. doi: 10.3390/ijms21239292 (PMC7730096; doi:10.3390/ijms21239292)
Supplement: Supplementary file 1 [file ijms-21-09292-s001.pdf]

International Journal of *Molecular Sciences*

MDPI

Supplementary Information

Self-Assembly of pH-Labile Polymer Nanoparticles for Paclitaxel Prodrug Delivery: Formulation, Characterization, and Evaluation

Shani L. Levit [1], Narendar Reddy Gade [1], Thomas D. Roper [1], Hu Yang [1,2,3] and Christina Tang [1,*]

[1] Chemical and Life Science Engineering Department, Virginia Commonwealth University, Richmond, VA 23284, USA; levitsl@vcu.edu (S.L.L), g.narendarreddy@gmail.com (N.R.G), tdroper@vcu.edu (T.D.R), hyang2@vcu.edu (H.Y.)

[2] Department of Pharmaceutics, Virginia Commonwealth University, Richmond, VA 23298, USA; (H.Y.)

[3] Massey Cancer Center, Virginia Commonwealth University, Richmond, VA 23298, USA (H.Y.)

* Correspondence: ctang2@vcu.edu

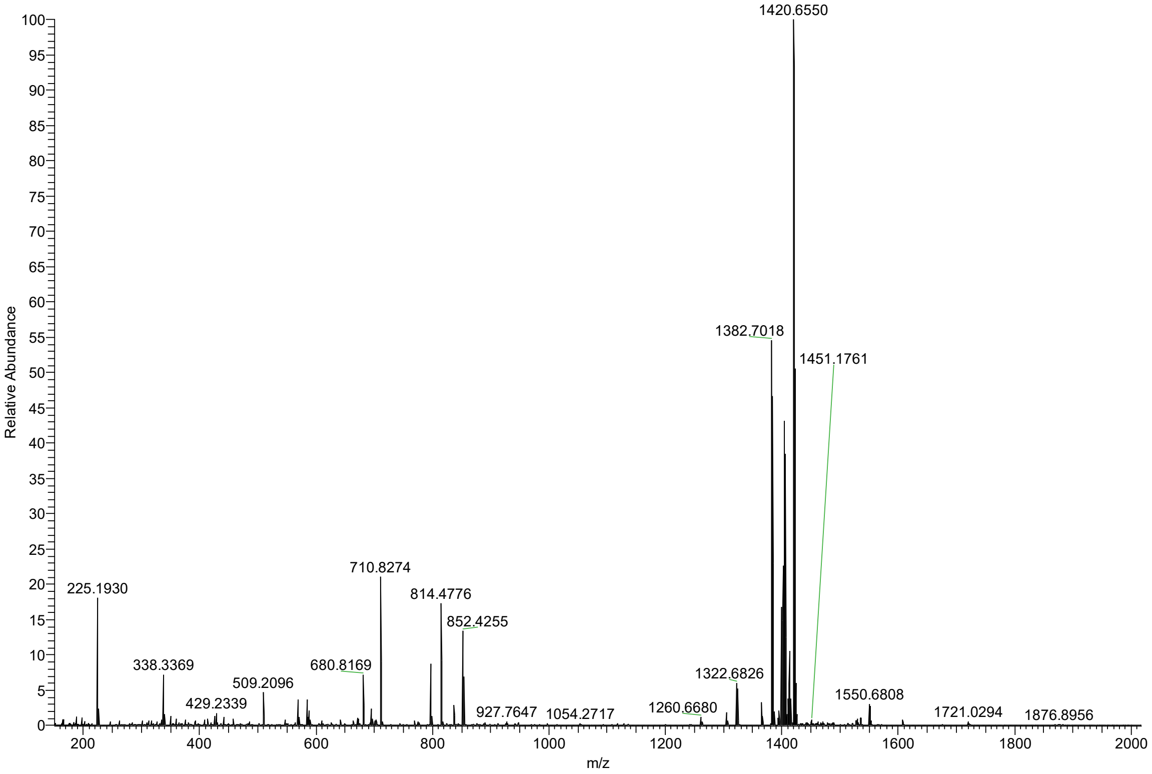

Figure S1. Direct Infusion Mass-Spectroscopy of the prodrug.

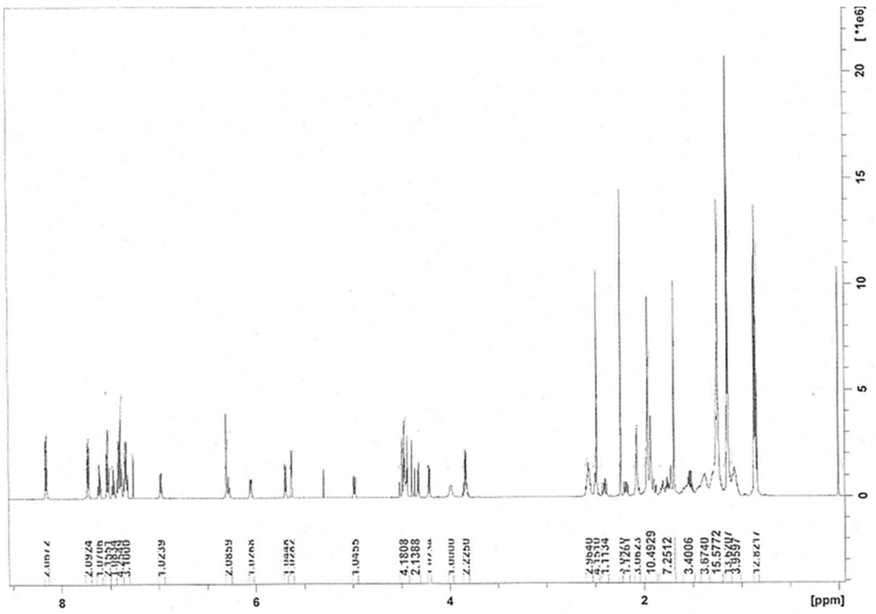

Figure S2. ^{1}H NMR of paclitaxel prodrug.

Table S1. The half maximal inhibitory concentration IC_{50} of ovarian cancer cell lineOVCA-432 cells treated with free paclitaxel (PTX and free paclitaxel prodrug (Pro).

Treatment	IC-50 (mM)
Free PTX	83 ± 6
Free Prodrug	10 ± 5

Table S2. Varying the concentration of the paclitaxel prodrug when formulating nanoparticles (Pro NPs). The formulation of paclitaxel nanoparticles (PTX NPs) was used as a starting point.

Sample	Ratio	Prodrug Concentration (mg/mL)	Size 1 (nm)	Size 2 (nm)	PDI
PTX NPs	2:1	1	111 ± 10	0	0.255 ± 0.021
	2:1	1	184 ± 11	26 ± 2	0.373 ± 0.050
Pro NPs	2:1	0.5	156 ± 18	29 ± 2	0.318 ± 0.021
	2:1	0.25	135 ± 6	-	0.206 ± 0.017

Table S3. Size stability of prodrug loaded nanoparticles (Pro NPs) in phosphate buffered saline (prepared at a 2:1 block copolymer:core ratio and prodrug concentration of 0.5 mg/mL).

	Initial			2 Months		
Pro NPs	Size 1 (nm)	Size 2 (nm)	PDI	Size 1 (nm)	Size 2 (nm)	PDI
	156 ± 18	29 ± 2	0.318 ± 0.021	178 ± 8	38 ± 5	0.287 ± 0.010

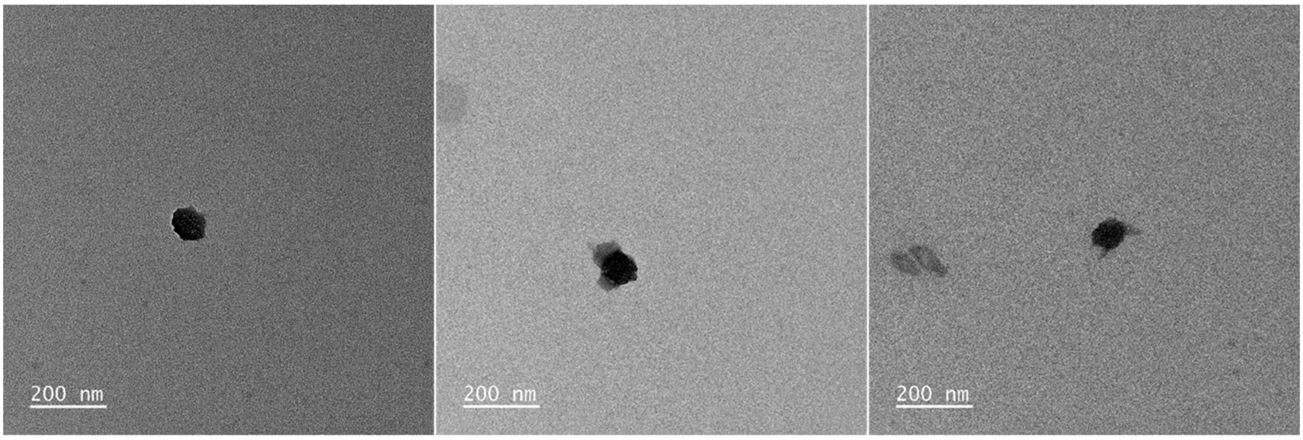

Figure S3. Transmission electron microscopy (TEM) images of prodrug-loaded nanoparticles (Pro NPs) (image in the manuscript provided on the left for comparison).

Table S4. Diffusion exponent (n) and coefficient of determination (R^2) of nanoparticle drug release at pH 4.0 conditions fit to the Korsemeyer-Peppas diffusion model.

Sample	Diffusion Exponent (*n*)	R^2
PTX NPs	0.26	0.80
Pro NPs	0.14	0.41

Table S5. Varying the BCP: core ratio of Pro-LAP NPs.

Sample	BCP: Core Ratio	Drug Concentration (mg/mL)		Size 1 (nm)	Size 2 (nm)	PDI
		Prodrug	LAP			
Pro-LAP NPs	2:1	0.5	0.5	169 ± 11	31 ± 3	0.361 ± 0.034
	1:1	0.5	0.5	145 ± 2	0	0.111 ± 0.018

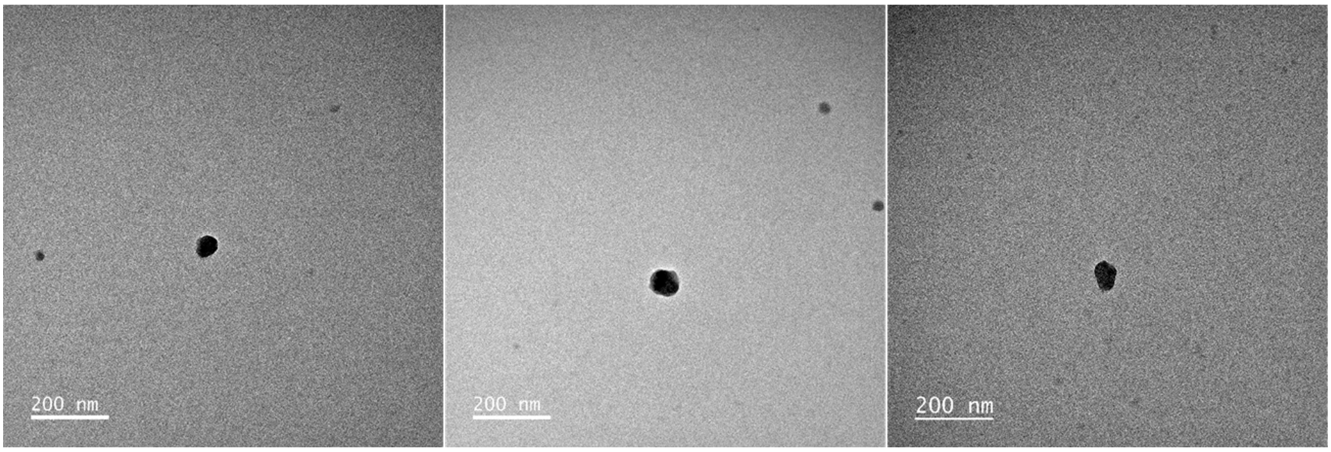

Figure S4. Transmission electron microscopy TEM images of prodrug/lapatinib-loaded nanoparticles (Pro-LAP NPs).

Table S6. Summary of the encapsulation efficiency (EE%) and drug loading (DL%) of prodrug/lapatinib-loaded nanoparticles Pro-LAP NPs.

Samples	Encapsulation efficiency (EE%)		Drug loading (DL%)	
	Prodrug	LAP	Prodrug	LAP
Pro-LAP NPs	38 ± 2	27 ± 11	1.01 ± 0.02	0.55 ± 0.15

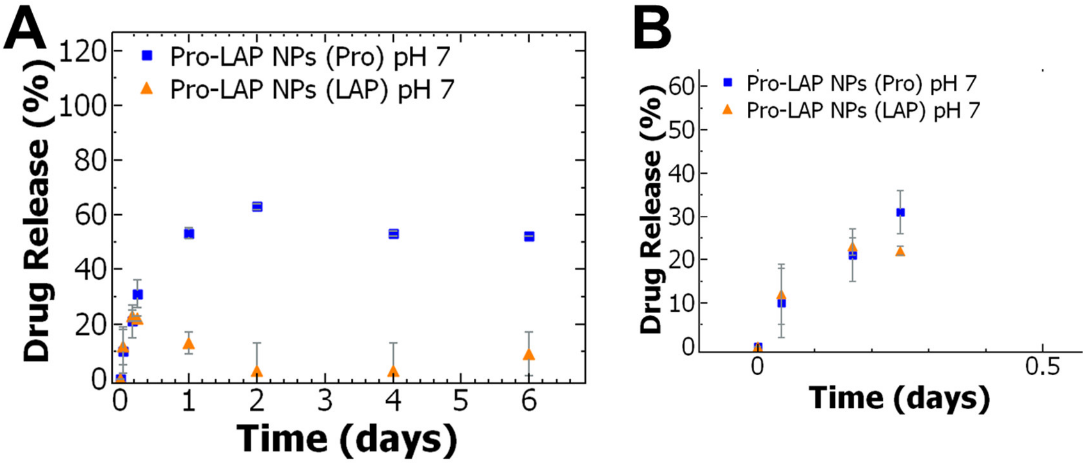

Figure S5. (**A**) The drug release profile of (blues) prodrug (Pro) and (orange) lapatinib (LAP) from co-loaded nanoparticles (Pro-LAP NPs) at pH 7 with a closer view of short times between 0 and 0.5 days shown in (**B**) ($n = 3$, error bars represent standard deviation of the 3 trials) The apparent in cumulative release may be attributed to supersaturation of the dialysis media.

Table S7. Diffusion exponent (n), rate constant (a), and coefficient of determination (R^2) of co-loaded nanoparticle drug release at pH 7.4 fit to the Korsemeyer-Peppas diffusion model.

Nanoparticle Samples	Drug	Diffusion Exponent (*n*)	Rate Constant (*a*)	R^2
Pro-LAP NPs	Prodrug	1.0	1.4	1.0
	LAP	1.0	2.3	1.0

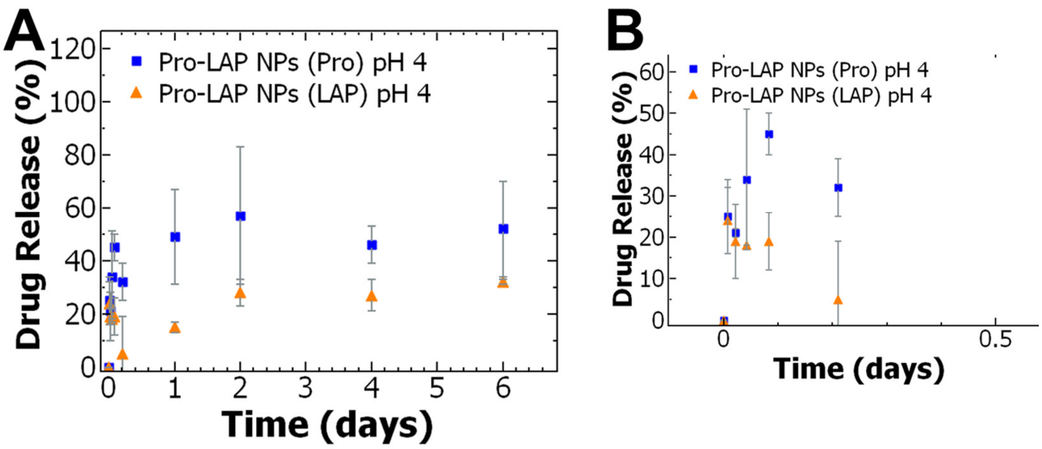

Figure S6. (**A**) The drug release profile of (blue) prodrug (Pro) and (orange) lapatinib (LAP) from co-loaded nanoparticles (Pro-LAP NPs) at pH 4 with a closer view of short times between 0 and 0.5 days shown in (**B**) ($n = 3$, error bars represent standard deviation of the 3 trials). The apparent in cumulative release may be attributed to supersaturation of the dialysis media.

Table S8. Rate constants (K_s) and coefficient of determination (R^2) of co-loaded nanoparticle drug release at pH 4 fit to the Hixson-Crowell diffusion model.

Nanoparticle Samples	Drug	Burst Release		Sustained Release	
		Rate Constant (K_S)	R^2	Rate Constant (K_S)	R^2
Pro-LAP NPs	Prodrug	3.1	0.99	0.049	0.73
	LAP	1.6	0.70	0.017	0.95
